# Editorial: Integrative advances in understanding and treating fears and phobias

**DOI:** 10.3389/fpsyg.2025.1674697

**Published:** 2025-09-16

**Authors:** Shakiela K. Davies

**Affiliations:** ^1^University of South Wales, Treforest, United Kingdom; ^2^University of South Wales, Pontypridd, United Kingdom

**Keywords:** fears, phobias, VRET, psychopathology, treatment, disgust, blood injection injury

An especially prominent and consequential area within psychopathology, both in terms of its widespread prevalence and the persistent uncertainties concerning its origins and effective therapeutic approaches, is the investigation of fears and phobias. Traditional aetiological research has attributed learned responses arising from early distressing childhood experiences. However, recent interdisciplinary research—ranging from behavioral, cognitive, physiological, and evolutionary methods—reveals that the mechanisms underpinning fear and phobias is far more complex. Notably, treatment interventions have advanced well beyond classical *in vivo* exposure, with Virtual Reality Exposure Therapy (VRET) and emerging immersive platforms providing more recent therapeutic forms of treatment management. Despite this progress, comparative empirical evidence evaluating virtual compared to real-world interventions remains scarce. This special editorial article provides an introduction and summary of four separate published studies (articles). The studies span across four prominent interrelated domains: *prevalence and demography; evolutionary and neurocognitive mechanisms; attentional processes and types of threat; and therapeutic advancement*. An introduction and summary of each article is provided below.

## Prevalence and demography

The emergence of smartphone use has contributed to a complex psychological dependence on mobile phone usage, resulting in nomophobia, defined as the significant distress caused by the absence of mobile phone connectivity. Mousoulidou et al. conducted a large-scale survey involving 300 adults in Cyprus to assess the prevalence and predictors of this phenomenon. The findings were clear: 99.3% of participants showed nomophobic symptoms, with 51.3% presented moderate levels. Younger age, female gender, and lower educational attainment emerged as significant risk factors, alongside social communication motives and maladaptive coping behaviors. These findings extend earlier research—primarily based on student samples—revealing how digital dependencies now constitute clinically salient anxieties across broader populations. Indeed, nomophobia has shown robust associations with comorbidities including social anxiety, insomnia, depression, smartphone addiction, and fear of missing out. These results call for the strategic integration of modern fear typologies into mental health practices and public health practices.

## Evolutionary and neurocognitive mechanisms—Fear and disgust

Moving from demographic/prevalence data toward human survival and threat-detection mechanisms, Landová et al. investigated the evolutionary bifurcation of threat systems—fear and disgust—within the Behavioral Immune System (BIS), positing disgust as its principal driver designed to identify and avoid pathogen exposure. Landová et al. conducted a survey of 60 vignettes related to fear and disgust associated with three forms of threat: (1) ancestral, (2) modern, and (3) airborne. The study showed that fear is largely attributed to modern threats such as electricity and car accidents, while disgust is predominantly elicited by ancestral stimuli such as bodily waste and worms. Interestingly, modern hazards (e.g., toxic chemicals) failed to evoke disgust, but elicited fear and anger. The findings also demonstrated that sensitivity to disgust dissipated with age, however fear presented as a compensatory mechanism for contamination avoidance. Meaning that the brain appears to transfer the emotional processing of disgust to fear as way to protect aging adults from potential contamination.

## Attentional processes and types of threat—Snakes and blood-injury-injection stimuli (BII)

Previous research on visual threat processing has predominantly overlooked the differences between types of threat, despite findings of category specific neural responses for certain fears. Zsido and Kiss investigated visual attentional effects of two types of threat—snakes and blood-injury-injection (BII)—presented as task-irrelevant distractors, whilst participants performed a visual search task. Findings showed that BII-related distractor pictures delayed reaction times compared to snake pictures. A key finding showed that those participants who used emotional regulation strategies showed enhanced performance in overriding the interference of threat stimuli on visual attention. The implications of emotional regulation and individual differences in threat perception and attention is highlighted.

## Therapeutic advancement

Therapeutic advancement, specifically via Immersive Virtual Reality (iVR), represents a key focus for future research on anxiety disorders. Findings on VRET (Virtual Reality Exposure Therapy) for acrophobia (severe anxiety when one is close to heights) confirms symptom reduction equivalent to that achieved through *in vivo* exposure—so long as interventions are therapist-led. Yet, suboptimal immersion, absence of biofeedback, and limited human presence impair efficacy. Varšová and Jurík argue that Collaborative Immersive Virtual Environments (CIVEs)—multiuser VR systems—offer promise by reintroducing essential therapist-patient interaction into the simulated context. VRET has shown to be a promising effective intervention for the treatment of acrophobia.

Taken together, the articles in this special edition highlight four converging themes:

- Prevalence and demographics—Nomophobia is an emergent digitally-driven fear that is becoming rapidly prevalent and requires further research and tailored interventions.
- Evolutionary: Disgust and Fear—are underpinned by BIS and other specialized neural circuits.
- Attentional processes and types of threat—Snakes and blood-injury-injection stimuli (BII)—specific forms of threat may trigger distinct behavioral responses highlighting the need for consideration of individual differences in anxiety, disgust and emotional regulation within this field.
- Therapeutic advancement—VRET offers a promising middle ground between traditional therapy and scalable digital interventions. Enhanced immersion, human presence, and biofeedback integration—especially within CIVEs—are key to maximizing therapeutic outcomes and adoption.

This special edition sets forth several key imperatives. First, systematically monitoring emerging fear types—such as nomophobia—and evaluating their psychosocial correlates is essential for early detection and targeted prevention. Second, comparative mechanistic research must elucidate the neural and cognitive fingerprints of different fear domains to guide more specialized interventions. Third, BIS theory should be further integrated into psychopathology, particularly in relation to disgust-driven pathology and its environmental modulators. Lastly, robust, longitudinal trials comparing *in vivo*, VRET, and CIVE-enhanced exposure—equipped with rigorous measures of immersion, physiological arousal, and long-term efficacy—are necessary to translate technological potential into clinical standard.

In conclusion, this editorial underscores the need for the future study of fears and phobias: one that speaks to evolving epidemiology, considers evolutionary-mechanistic underpinnings, and prioritizes tailored interventions. By weaving these strands together, it aims to enrich both theoretical insight and evidence-based clinical practice—charting a path for research and innovation in an era of rapid technological change.

